# Analysis of Genomic and Characterization Features of *Microbulbifer weihaiensis* sp. nov., Isolated from Coastal Sediment

**DOI:** 10.3390/microorganisms13092005

**Published:** 2025-08-28

**Authors:** Yu-Xuan Zhang, Ai-Qiu Liu

**Affiliations:** 1 SDU-ANU Joint Science College, Shandong University, Weihai 264209, China; 13021511137@163.com; 2 Marine College, Shandong University, Weihai 264209, China

**Keywords:** *Microbulbifer weihaiensis* sp. nov., 16S rRNA gene, comparative genomic analysis, biogeographic distribution

## Abstract

A strictly aerobic, straight-rod, motile Gram-negative bacterium, SDUM041083^T^, was isolated from marine sediment in Xiaoshidao, Weihai, China, in the formation of yellowish-brown colonies. Its growing conditions are as follows: 20–40 °C, pH 5.5–9.5, and 0.5–11% (*w*/*v*) NaCl. Phylogenetic analysis of the 16S rRNA gene sequence showed that SDUM041083^T^ was related to members of the genus *Microbulbifer*. Strain SDUM041083^T^ showed the highest 16S rRNA gene sequence similarity (98.23%) with *Microbulbifer okinawensis* JCM 16147^T^. The primary cellular fatty acids of SDUM041083^T^ were iso-C11:0 3-OH, iso-C11:0, and iso-C15:0. The respiratory quinone of SDUM041083^T^ was Q-8, and the polar lipids were phosphatidylglycerol, phosphatidylethanolamine, and one aminolipid. The genomic DNA G+C content of SDUM041083^T^ was 57.5 mol%. The phenotypic and genotypic characteristics of SDUM041083^T^ indicate that the strain should be classified as a new species representing the genus *Microbulbifer*, with the name *Microbulbifer weihaiensis* sp. nov. being proposed. The type strain was SDUM041083^T^ (=KCTC 8896^T^ = MCCC 1H01537^T^). Comparative genomic analysis showed that the 32 *Microbulbifer* species shared 1446 core genes and differed mainly in terms of lipid metabolism, signal transduction and xenobiotic biodegradation and metabolism. Preliminary research showed that SDUM041083^T^ has the potential to degrade chitin. Biogeographic distribution analysis showed that the marine environments constitute the main habitat of the genus *Microbulbifer*.

## 1. Introduction

Marine ecosystems harbor a vast diversity of microorganisms that play pivotal roles in global biogeochemical cycles, particularly in the degradation and recycling of complex organic matter. The genus *Microbulbifer* allocated to the class *Gammaproteobacteria* was first proposed by González et al. (1997) [1] as a strictly aerobic Gram-negative bacterium capable of utilizing a variety of hydrocarbons [1]. At the time of writing this paper, the genus already contains 36 species with validly published names at https://lpsn.dsmz.de/genus/microbulbifer (accessed on 25 March 2025). Other than *Microbulbifer halophilus* from the saline soil of the Ganjiahu Natural Reserve [2] and *Microbulbifer rhizosphaerae* from the rhizosphere of a halophytic plant [3], the majority of *Microbulbifer* members were isolated from diverse marine and saline environments, such as tidal flat [4], deep-sea sediment [5], intertidal sediment [6], estuarine sediment [7], marine solar saltern [8], the surfaces of algae [9], sponge [10] and mangrove sediment [11,12,13]. Members of the *Microbulbifer* genus are characterized as Gram-stain-negative, some with sliding properties and some with single or multiple flagella [5,14].

Their extensive distribution and adaptive capabilities enable them to degrade a wide array of recalcitrant polysaccharides, including agar, carrageenan, alginate, chitin, cellulose, xylan, and pectin [15]. More than half of the *Microbulbifer* species exhibit amylase activity, as exemplified by *Microbulbifer aggregans* JCM 31875^T^ [7]. Additionally, several strains, including *Microbulbifer agarilyticus* JCM 14708^T^ [5] and *Microbulbifer thermotolerans* JCM 14709^T^ [5], possess the capability to degrade agar. Moreover, chitin degradation has been observed in strains such as *Microbulbifer okinawensis* JCM 16147^T^ [11]. Additionally, numerous studies have demonstrated that certain strains within this genus, such as *Microbulbifer elongatus* [14] and *Microbulbifer mangrovi* [12], possess alginate degradation pathways. Notably, *Microbulbifer mangrovi* [12] is a bacterium with remarkable polysaccharide-degrading capabilities, capable of breaking down 11 types of polysaccharides, including chitin, agar, pectin, and carrageenan. This enzymatic characterization positions the strains of *Microbulbifer* as key players in marine carbon cycling, particularly in the breakdown of macroalgal biomass and other complex biopolymers that are inaccessible to many other microorganisms. Many polysaccharide enzymes (e.g., agarases, carrageenases, chitinases, cellulases) possess unique properties such as salt tolerance [16], thermal stability [17], and optimal alkaline pH [17], making them ideal candidates for industrial processes in biofuel production, food processing, textile manufacturing, and waste bioremediation. Additionally, several *Microbulbifer* strains demonstrate capabilities in the bioremediation of pollutants such as hydrocarbons [1], polyhydroxybutyrate (a biodegradable plastic) [18,19], and polyethylene [20], highlighting their applicability in environmental cleanup.

Moreover, the genus is a promising source of bioactive compounds; 32 different natural compounds have been isolated from this genus [15], including 4-hydroxybenzoate [21] and alkaloids [22]. The rich biosynthetic gene clusters in the genome will lead to the discovery of new natural chemical products in the future.

With the maturation of new bacterial identification techniques, an increasing number of strains from the genus *Microbulbifer* have been discovered. However, there are few studies analyzing the genomic comparisons [23] and geographical distributions of this genus. During research into the diversity of culturable marine bacteria found in Xiaoshidao, Weihai, China, a bacterial strain forming yellow-brown colonies, named SDUM041083^T^, was isolated, representing a potential new species of the genus *Microbulbifer*. The strain representing a new species of the genus *Microbulbifer* isolated from shoal sediments is characterized in this study.

## 2. Materials and Methods

### 2.1. Bacterial Isolation and Cultivation

Strain SDUM041083^T^ was isolated from shoal sediments collected from Xiaoshi Island, Weihai, China (37.5° N, 122.1° E) in 2023. The samples were diluted with sterile seawater in five gradients using a standard dilution-plating technique [24], and 0.1 mL aliquots of each dilution were uniformly coated on marine agar 2216 (MA; Becton Dickinson, Franklin Lakes, NJ, USA) and incubated at a constant temperature of 28 °C in an incubator. Single colonies were separated from MA for identification, and finally, the new species SDUM041083^T^ was isolated and stored in sterile 20% (*v*/*v*) glycerol supplemented with 3% (*w*/*v*) NaCl at −80 °C. The reference strain was identified by 16S rRNA gene sequence comparisons and phylogenetic trees as *Microbulbifer okinawensis* [11], which can grow on MA media.

### 2.2. 16S rRNA Gene Sequencing and Phylogenetic Analysis

The 16S rRNA gene of the strain was amplified by PCR using universal primers 27F and 1492R [25]. Pairwise comparisons of 16S rRNA gene sequences were carried out using the NCBI [National Centre for Biotechnology Information (nih.gov)] databases [26] and EzBioCloud (https://www.ezbiocloud.net/, accessed on 10 April 2025) [27]. Phylogenetic analyses were carried out using maximum parsimony (MP) [28], neighbor joining (NJ) [29], and maximum likelihood (ML) [30] methods, as well as the software package MEGA version 11.0 [31,32]. For the NJ algorithm, genetic distances were calculated using the Kimura two-parameter model [33]. The generated tree topologies were evaluated by bootstrap analysis based on 1000 replicates.

### 2.3. Whole-Genome Sequencing and Genomic and Phylogenomic Analyses

The genomic DNA of SDUM041083^T^ was extracted and purified using the Bacterial Genomic DNA Mini Kit (Takara Bio, Kusatsu, Japan) according to the manufacturer’s instructions. The draft genome of strain SDUM041083^T^ was sequenced using the NovaSeq 6000 platform (Illumina) by Beijing Novogene Bioinformatics Technology (Beijing, China). The processing steps were as described by Wang et al. [34]. Genomic data for the remaining strains in this research were obtained from the NCBI genome repository.

Genome annotation was carried out using the NCBI Prokaryotic Genome Annotation Pipeline (PGAP), which relies on initial gene prediction algorithms and homology-based approaches [35]. The secondary metabolite biosynthetic gene clusters within the SDUM287046^T^ strain genome were examined using the online antiSMASH server (version 7.0; https://antismash.secondarymetabolites.org/, accessed on 3 April 2025) [36].

To define the exact taxonomic position, the genome-wide average nucleotide identity (ANI) and average amino acid identity (AAI) were estimated using the JSpeciesWS online service (https://jspecies.ribohost.com/jspeciesws/, accessed on 2 April 2025) [37] and the AAI calculator (http://enve-omics.ce.gatech.edu/aai/, accessed on 3 April 2025), respectively. Digital DNA-DNA hybridization (dDDH) of whole genome sequences between strains was carried out utilizing DSMZ’s online service (http://ggdc.dsmz.de, accessed on 3 April 2025) [38]. Pathway analysis was performed on the KEGG website (https://www.genome.jp/kegg/, accessed on 16 April 2025) [39]. The concatenated alignment sequences of 120 ubiquitous single-copy proteins were obtained by GTDB-Tk (version 2.4.0) [40], and the phylogenetic tree was reconstructed by using the JTT + CAT parameters in FastTree [41] and the LG + F + I + G4 model with 1000 bootstrap replicates in IQ-TREE [42].

### 2.4. Comparative Genomic Analysis and Biogeographic Distribution of the Genus Microbulbifer

KEGG’s BlastKOALA (https://www.kegg.jp/blastkoala/, accessed on 18 April 2025) [43] server was utilized to thoroughly examine the metabolic routes of all genomes. To estimate the genomic diversity and identify orthologous groups among the species of the genus *Microbulbifer*, pan-genome analysis was performed using the bacterial pan-genomic analysis (BPGA) tool, with default parameters (50% amino acid sequence identity) [44].

Given that previous studies on *Microbulbifer* reported superior activities for polysaccharide hydrolysis or degradation, the CAZy (carbohydrate-active enzymes) database (https://www.cazy.org/, accessed on 3 May 2025) [45] and the dbCAN2 web server with default parameters (https://bcb.unl.edu/dbCAN2, accessed on 5 May 2025) were used to investigate the polysaccharide hydrolysis or degradation activities of SDUM041083^T^.

The analysis pipeline Microbe Atlas Project (MAP, https://microbeatlas.org/, accessed on 22 April 2025) was used to assess the global distribution and habitat preferences of the genus *Microbulbifer*. In this study, a strict 96% sequence similarity threshold was adopted. Microbial community abundance was determined using MAPseq, a closed reference method for the analysis of ribosomal RNA sequences [46].

### 2.5. Morphology, Physiology, and Biochemistry

The morphology of the cell was observed by light microscopy (E600, Nikon, Tokyo, Japan) and field emission scanning electron microscopy (Nova NanoSEM 450, FEI, Portland, OR, USA). To facilitate subsequent experiments, the temperature, salinity, pH ranges, and optimum conditions of the strains were first tested. To determine its growth temperature range and optimal temperature, SDUM041083^T^ was cultured on MA medium across 11 temperature gradients (4, 10, 20, 25, 28, 30, 33, 35, 37, 40, and 48 °C). Specifically, each MA plate was streak-inoculated via three-zone streaking with uniformly sized single colonies from pre-cultured SDUM041083^T^, with three replicate plates per temperature. Plates were observed every 12 h to record the emergence time of colonies in Zone 1 and Zone 3. For replicates with time differences ≤24 h, the mean value was used; differences > 24 h (not observed here) would have required re-testing. The optimal growth temperature was defined as the temperature with the shortest mean emergence time of Zone 3, while the growth temperature range included all temperatures where Zone 1 colonies appeared. To detect the growth salinity, MA was prepared with artificial seawater, and after removing the original NaCl, the salinity was divided into 11 gradients (0%, 1%, 2%, 3%, 4%, 5%, 6%, 7%, 8%, 9%, and 10%) by adding different concentrations of NaCl to then be placed in a constant temperature incubator at 33 °C simultaneously for cultivation, using the same observation method as the temperature. The experiment was repeated, and the salinity gradients were increased to 15 (0.5%, 1%, 1.5%, 2%, 2.5%, 3%, 3.5%, 4%, 5%, 7%, 10%, 11%, 12%, 13%, and 14%). Zhu et al.’s approach was used to identify the pH range in which it can grow and its optimum pH [25]. OD_600_ measurements were analyzed to determine the growth range by identifying pH values where statistically significant and sustained increases in biomass occurred. The optimal pH was determined by integrating both maximum biomass accumulation and growth rate metrics across the pH gradient. The Gram staining was tested using a Hopebio Gram-stain Kit (HB8278-2, Qingdao Hope Biotechnology, Qingdao, China). Nitrate reduction is determined as follows: Two MA mediums were prepared—one was the normal version, while the other MA medium was added with 0.1% (*w*/*v*) KNO_3_. The strain was cultured on MA with and without KNO_3_ and then incubated in an anaerobic bag for 15 days to test the growth in anaerobic conditions, while the other group was not placed in an anaerobic bag, being incubated normally in an incubator. SDUM041083^T^ was found on an MA semisolid medium supplemented with 0.3% agar and observed for gliding movements, as described by Bowman et al. [47]. The degradation of Tween (including Tween 20, 40, 60, and 80), casein, carboxymethylcellulose, sodium alginate, and agar was tested on MA plates supplied with the corresponding substrates [48]. For the preparation of chitinase activity assay plates, colloidal chitin (0.8% *w*/*v*) was employed as the sole carbon source in a medium formulated with artificial seawater and ammonium sulfate. The pH of the medium was adjusted to 7.0 ± 0.2 using a phosphate buffer system (potassium hydrogen phosphate/potassium dihydrogen phosphate). Single colonies of strain SDUM041083^T^ and reference strains were inoculated onto these plates, with three replicates per strain, and were incubated at their optimal growth temperature of 35 °C for 7 days; the hydrolysis zone-to-colony diameter ratio (H/C) was calculated for each strain, while Welch’s *t*-test was used to assess chitinolytic activity. The DNAase activity was tested using DNAase agar (HB4118, Hope Bio-Technology Co., Ltd., Qingdao, China) supplemented with 3% (*w*/*v*) NaCl. Oxidase activity and catalase activity were tested according to Wang et al. [34]. The disk diffusion method was used to investigate antibiotic sensitivity on MA as described by Wang et al. [49]. Additional physiological or biochemical characterization was carried out using API 20E, API ZYM, and API 50CHB test strips (all from BioMérieux, Shanghai, China) and GEN III microtiter plates (Biolog, Hayward, CA, USA) according to the manufacturer’s instructions.

### 2.6. Chemotaxonomy

For the analysis of cellular fatty acids, polar lipids, and isoprenoid quinones, both SDUM041083^T^ and the reference bacterium were incubated in an MA medium at 33 °C for 48 h. The determination of fatty acids and the processing of the results were carried out in full accordance with the steps of Liang et al. [50]. Silica gel TLC plates (Kieselgel 60 F254; Merck, Rahway, NJ, USA) were used to analyze the quinone types, and the content of each quinone type was subsequently analyzed by HPLC as described previously [51]. Polar lipids were analyzed according to the method of Tindall et al. [52]. Molybdenum phosphate was employed to detect total lipids, whereas spray reagents were used to identify the various functional groups. For the detection of respiratory quinone, the organisms were collected to make lyophilized powder, and the assay was performed as described by Liu et al. [53].

## 3. Results and Discussion

### 3.1. Phenotypic Properties

The cells of strain SDUM041083^T^ were Gram-negative and aerobic, measuring 4.780 ± 0.2 μm and 0.326 ± 0.1 μm in length and in width, respectively (Figure S1). The optimal growth temperature for strain SDUM041083^T^ was 35 °C, with a growth temperature range of 20–40 °C. It was observed that a 3.5% NaCl concentration is the optimal salinity for the growth of SDUM041083^T^, similar to *Microbulbifer okinawensis* [11]. The bacterium could grow at salinities from 0.5 to 11%, although growth was very slow at 7–11%. It grows across a pH range of 5.5 to 9.5, with an optimal pH of 7.0. Both the range and optimal pH are comparable to those of *Microbulbifer okinawensis* [11] and *Microbulbifer yueqingensis* [54]. The bacterium is motile despite lacking flagella; some strains in this genus, such as *Microbulbifer thermotolerans* [5] and *Microbulbifer elongatus* [14], are also motile. According to the manufacturer’s instructions, Biolog GENIII microplate reagent strips were used to assess the substrate utilization of the isolated bacterium SDUM041083^T^. After five days, 58 substrates tested positive, including D-Maltose, D-Cellobiose, Sucrose, N-Acetyl-D-Glucosamine, N-Acetyl-β-D-Mannosamine, N-Acetyl-D-Galactosamine, Gelatin, L-Arginine, L-Histidine, Tween-40, α-D-Glucose, D-Mannose, D-Fructose, and D-Galactose, among others. The API ZYM test results indicated positive activity for lipase (C14) and N-acetyl-glucosaminase (Table 1).

Strain SDUM041083^T^ exhibited a lack of alginate lyase, cellulase, agarase, and DNAase activities. It utilized Tween 20, 40, 60, and 80 and displayed both tyrosinase and catalase activities. In the chitinase activity assay, hydrolysis zones appeared around colonies after 7-day incubation at 35 °C (Figure S2). Strain SDUM041083^T^ demonstrated a hydrolysis zone-to-colony diameter ratio (H/C) of 4.89 ± 0.30, with the hydrolysis zone and colony diameter being 1.03 ± 0.14 cm and 0.22 ± 0.04 cm, respectively. In contrast, the reference strain *M. okinawensis* JCM 16147^T^ had a significantly lower H/C ratio of 2.84 ± 0.03, with a hydrolysis zone and colony diameter of 2.37 ± 0.07 cm and 0.83 ± 0.03 cm, respectively (Figure S2). Notably, the H/C ratio of SDUM041083^T^ was significantly higher than that of *M. okinawensis* JCM 16147^T^ (* *p* < 0.05). This implies that despite its constrained apparent hydrolytic coverage (smaller absolute zones), SDUM041083ᵀ shows superior chitinolytic efficacy per unit biomass.

In addition, SDUM041083^T^ displayed resistance to lincomycin (2 μg), tetracycline (30 μg), vancomycin (30 μg), penicillin (10 μg), streptomycin (10 μg), ceftriaxone (30 μg) and kanamycin (30 μg).

### 3.2. Chemotaxonomic Characteristics

The main fatty acids of strain SDUM041083^T^ were iso-C_15:0_ (24.33%), iso-C_11:0_ 3-OH (10.78%), and iso-C_11:0_ (10.61%) (Table S1), which shared a major fatty acid iso-C_15:0_ with other *Microbulbifer* species, such as *Microbulbifer okinawensis* [11], *Microbulbifer marinus* [54], *Microbulbifer taiwanensis* [56], and *Microbulbifer bruguierae* [23], and were the same as those of *Microbulbifer yueqingensis* [54]. Among them, only C_11:0_ was not the main fatty acid of the reference bacterium (Table S1). The respiratory quinone of SDUM041083^T^ was Q-8, which is consistent with the reference bacterium [11]. The polar lipids of strain SDUM041083^T^ were phosphatidylglycerol (PG), phosphatidylethanolamine (PE), and Aminolipid (AL) (Figure S3), which were likewise identical to the reference bacteria in this experiment.

### 3.3. The 16S rRNA Gene Sequence and Phylogenetics

**Figure 1:**
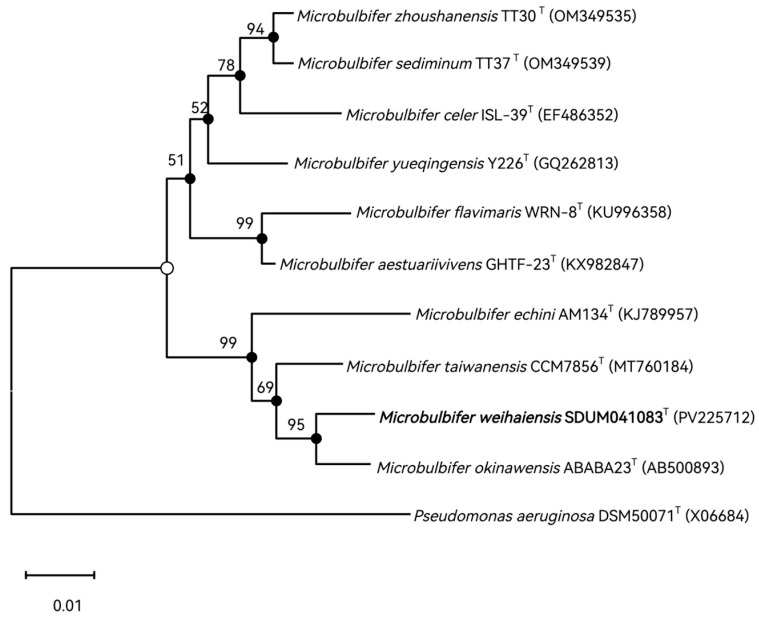
Phylogenetic tree reconstructed using MEGA based on 16S rRNA gene sequence data, showing the phylogenetic position of strain SDUM041083^T^ among related taxa. GenBank accession numbers of 16S rRNA gene sequences are given in parentheses. Numbers on nodes represent bootstrap values based on 1000 replications. The strain characterized in this study is shown in bold type. Filled circles indicate that the corresponding nodes were also recovered in maximum-likelihood and maximum-evolution analyses. *Pseudomonas aeruginosa* DSM50071^T^ was used as the outgroup. Open circles indicate that the corresponding nodes were also recovered in either the maximum-likelihood or the minimum-evolution analyses. Bar, 0.02 substitutions per nucleotide position.

The 16S rRNA gene sequence of strain SDUM041083^T^ (1415 bp) was aligned with the EzBioCloud database and NCBI. Strain SDUM041083^T^ has the highest similarity to *Microbulbifer okinawensis* (98.23%). Phylogenetic analysis of 16S rRNA gene sequences revealed that SDUM041083^T^ was grouped with members of the genus *Microbulbifer* and formed a coherent cluster, indicating that it might be considered a novel representative of the genus *Microbulbifer* (Figure 1). Comparable structural patterns of the SDUM041083^T^ strain and its related species were similarly observed in the phylogenetic trees reassembled using the ML and ME algorithms.

### 3.4. Genomic Features and Phylogenomics

The draft genome sequence of strain SDUM041083^T^ was assembled, showing that its DNA G+C content was 57.5 mol%, with a genome of 4,481,582 bp, generating 38 contigs and with a maximum length of 924,620 bp, an average length of 117,918.5 bp, an N50 value of 398,728 bp, and 3686 predicted gene sequences. Scaffolds of the draft genome sequences of strain SDUM041083^T^ were deposited in GenBank under the accession code JBLWFL000000000. According to the results of PGAP, the genome of strain SDUM041083^T^ contained 3760 genes, including 3696 protein-coding genes, 8 pseudogenes, and 56 RNA genes (3 rRNA, 49 tRNA, and 4 ncRNA).

**Figure 2:**
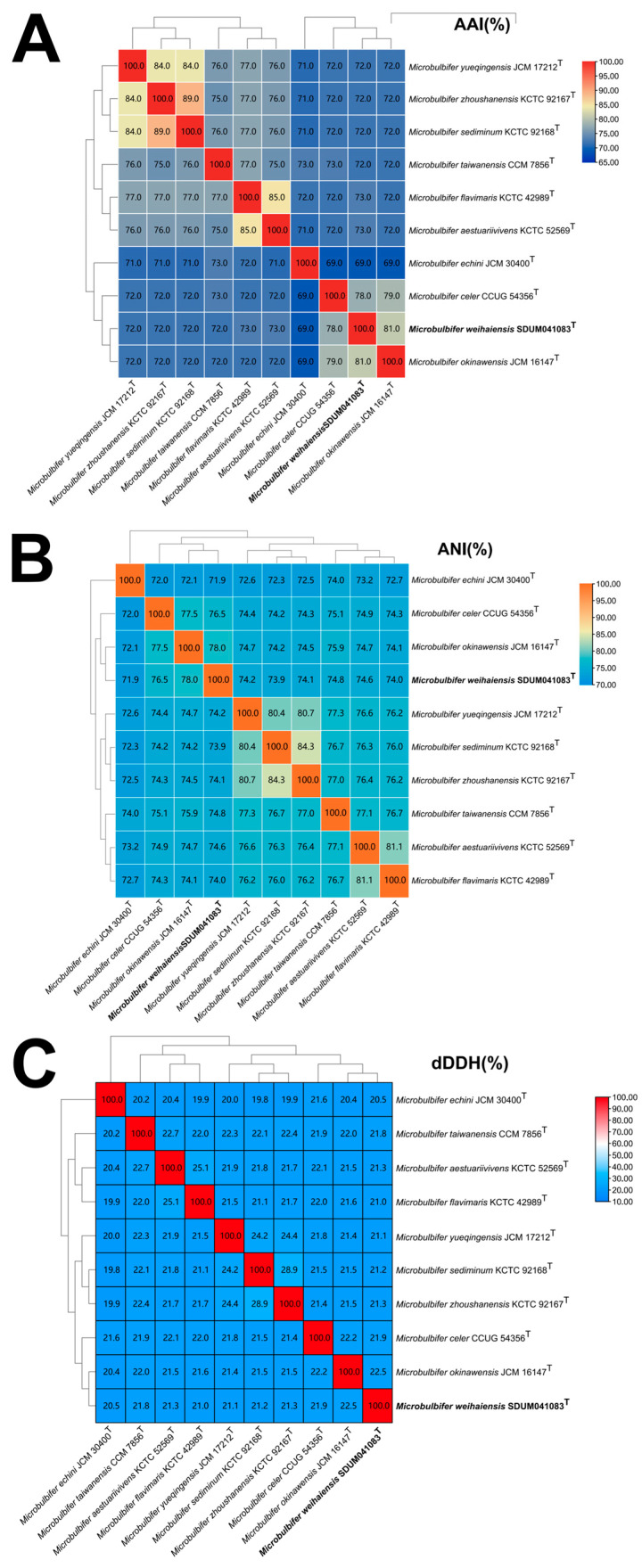
Genomic similarities of strain SDUM041083^T^ to some members of the genus *Microbulbifer*. (**A**) The AAI values between species of the genus. AAI: average amino acid identity. (**B**) The ANI values between species of the genus. ANI: average nucleotide identity. (**C**) The dDDH values between species of the genus. dDDH: digital DNA-DNA hybridization.

**Figure 3:**
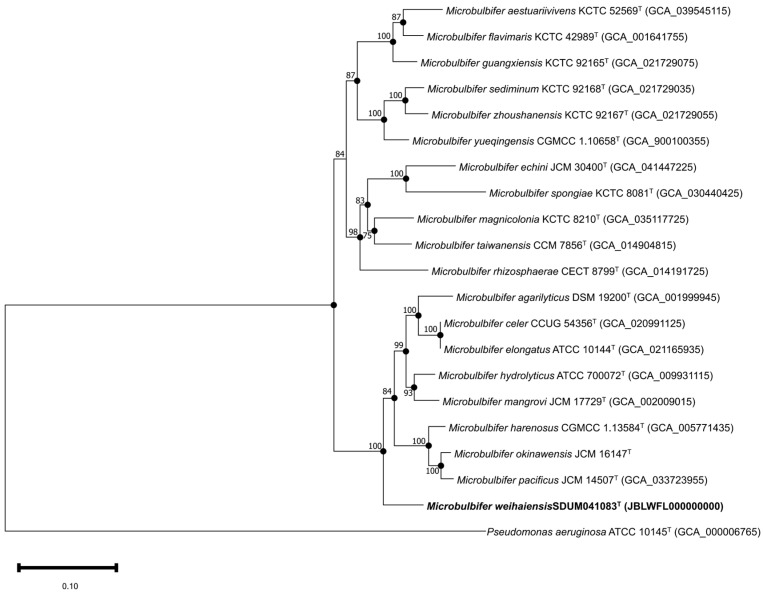
The FastTree is based on 120 ubiquitous single-copy proteins. Bootstrap values above 50% (1000 replicates) are shown at branch nodes. Filled circles indicate that the same topology is also obtained using the IQ-Tree algorithm. *Pseudomonas aeruginosa* ATCC 10145^T^ was used as the outgroup. Bar: 0.20 substitutions per nucleotide position.

Based on the anti-SMASH results, eleven secondary metabolite biosynthesis gene clusters were predicted in the genome of the SDUM041083^T^ strain, including gene clusters encoding non-ribosomal peptide synthetase-like fragments, mesophenol, Type I polyketide synthase, terpene-precursor, ectoine, non-ribosomal peptide synthetase, NRPS-independent IucA/IucC-like siderophores, terpene, aryl polyene, betalactone, and other unspecified ribosomally synthesized and post-translationally modified peptide products. The ANI value between strain SDUM041083^T^ and *Microbulbifer okinawensis* was 78.01%, which is below the 95% interspecies threshold [57]; the AAI value was 73.73% and the dDDH value was 22.50%, also below the threshold [58,59]. The ANI, AAI, and dDDH values of SDUM041083^T^ and other strains of the genus *Microbulbifer* were also lower than the threshold values (as shown in Figure 2), indicating that strain SDUM041083^T^ is a novel species of the genus *Microbulbifer*. The protein phylogenetic tree, which depicted the evolutionary relationships between strain SDUM041083^T^ and some related type strains, revealed that the strain was affiliated with the genus *Microbulbifer* (Figure 3), which was consistent with the result of the 16S rRNA gene phylogenetic analysis.

### 3.5. Comparative Genomic Analysis and Pan-Genome Analysis of the Genus Microbulbifer

**Figure 4:**
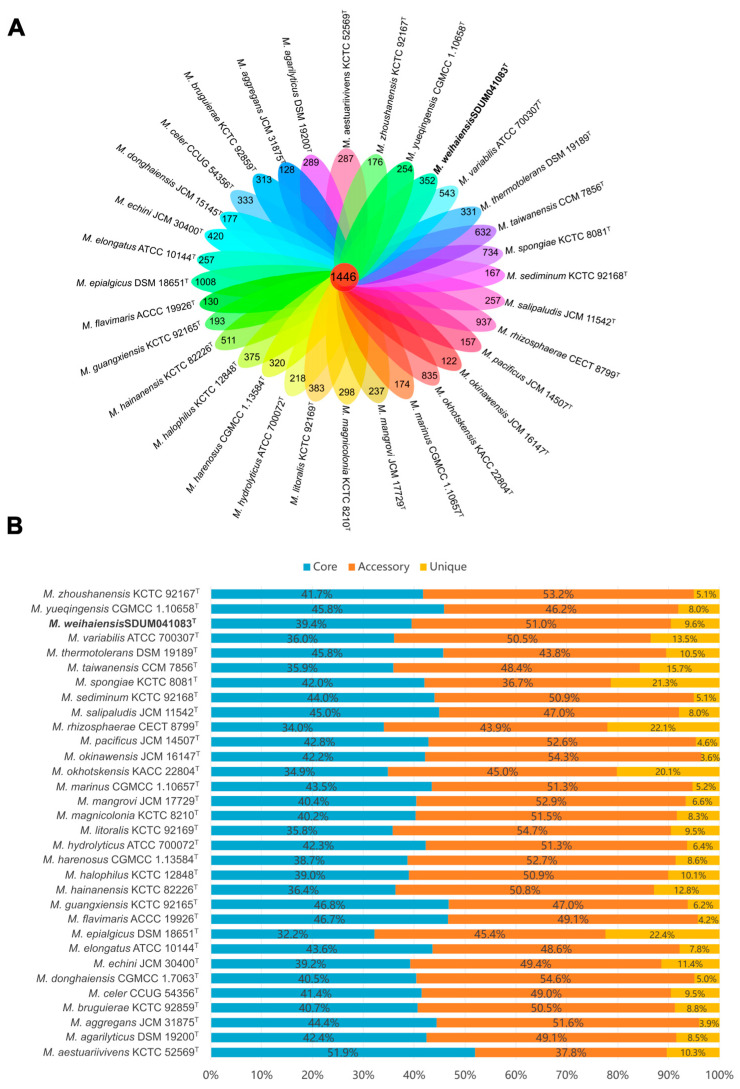
Pan-genome analysis of some strains of the genus *Microbulbifer.* (**A**) Venn diagram displaying the number of core gene families and unique genes for each strain. (**B**) Percentage of core, accessory, and unique genes in each genome.

The genome sizes of the genus, including strain SDUM041083^T^, ranged from 3.4 Mbp (*Microbulbifer aestuariivivens* GHTF-23^T^ [4]) to 5.7 Mbp (*Microbulbifer epialgicus* DSM 18651^T^ [9]). The genomic DNA G+C content varied from 49.0 mol% (*Microbulbifer variabilis* ATCC 700307^T^ [9] and *Microbulbifer epialgicus* DSM 18651^T^ [9]) to 62.0 mol% (*Microbulbifer yueqingensis* JCM 17212^T^ [54]). As illustrated in Figure 4, the pan-genome analysis based on orthologous groups of proteins revealed that 1446 core genes were shared by 32 *Microbulbifer* species, accounting for 32.2% to 51.9% of the genes in each genome (Figure 4A). The percentage of accessory genes and unique genes in each *Microbulbifer* genome ranged from 36.7% to 54.7% and from 3.6% to 22.4%, respectively (Figure 4B). The analysis of KEGG revealed that core genes predominantly contributed to essential metabolic pathways, including amino acid metabolism, cofactor and vitamin metabolism, nucleotide metabolism, replication and repair, and translation (Supplementary Figure S4). This result suggests a high degree of conservation in these cellular biological functions among the genus *Microbulbifer*. Additionally, accessory genes and unique genes were more distributed than core genes in carbohydrate metabolism, lipid metabolism, signal transduction, the metabolism of terpenoids and polyketides, and xenobiotic biodegradation and metabolism (Supplementary Figure S3), which indicated that these genes conferred metabolic diversity such as degrading a series of polysaccharides, producing a variety of natural products [15], degrading poly (3-hydroxybutyrate) (PHB) [18,19], and marine environment adaptability to the members of the genus *Microbulbifer* [60].

Notably, among the 32 analyzed strains, the genus *Microbulbifer* exhibits a broad genomic G+C content range (49.0–62.0 mol%) [9,54]. Such diversity may imply genomic variation; however, as mentioned above, all strains cluster into a well-supported clade in the protein phylogenetic tree (Figure 3), and the AAI, ANI, and dDDH values in Figure 2 all exceed the thresholds for defining a new genus, alongside partially conserved phenotypic traits (like aerobic metabolism and halotolerance). Additionally, pan-genome analysis further reveals 1446 core genes enriched in key pathways. For these reasons, it is tentatively maintained that they still belong to the single genus *Microbulbifer*. At the same time, it is preliminarily inferred that this G+C variation may stem from evolutionary processes, such as horizontal gene transfer (facilitated by the organic-rich habitats of this genus) and adaptive divergence into distinct ecological niches, while more accurate analysis will require further in-depth research in subsequent studies.

**Figure 5:**
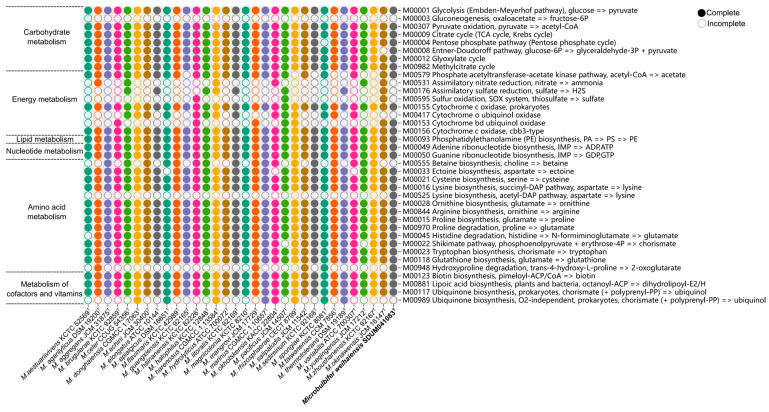
The metabolic module integrity of the genus *Microbulbifer*. The solid circles and hollow circles indicate that the metabolic pathways were complete and incomplete, respectively.

The metabolic pathways analyzed using KEGG’s BlastKOALA service of 32 *Microbulbifer* species (Figure 5) showed that the genus *Microbulbifer* demonstrated conservatism in carbohydrate metabolism, lipid metabolism, nucleotide metabolism, amino acid metabolism, and the metabolism of cofactors and vitamins, with some variations observed primarily in energy metabolism such as assimilatory nitrate reduction (M00531), assimilatory sulfate reduction (M00176), and sulfur oxidation (M00595). Specifically, in carbohydrate metabolism, all strains have complete key pathways like glycolysis (M00001), pyruvate oxidation (M00307), and TCA cycle (M00009). This conserved central metabolic network could efficiently process some monosaccharides derived from polysaccharide degradation into energy and biosynthetic precursors. Gluconeogenesis (M00003) was not complete in all 32 species of *Microbulbifer.* While central carbon metabolism exhibited broad conservation, *Microbulbifer. spongiae* KC 8081^T^ uniquely lacked a complete pentose phosphate pathway (M00004), suggesting the species–specific modulation of carbon flux. Regarding amino acids, all strains within the *Microbulbifer* genus exhibited completeness in most amino acid biosynthesis pathways, including ornithine biosynthesis (M00028), proline biosynthesis (M00015), Arginine biosynthesis (M00844), and lysine biosynthesis pathway (M00527). Notably, SDUM041083^T^ harbors a distinct lysine biosynthesis pathway (M00525), which is incomplete in other *Microbulbifer* strains. The lysine synthesis capacity of SDUM041083^T^ may provide a survival advantage in marine sediment environments. Additionally, proline [61] and arginine [62] have been shown to promote bacterial growth under hypertonic conditions by enhancing osmotic tolerance through intracellular amino acid accumulation [63]. The *Microbulbifer* genus’s strains possess multiple complete amino acid biosynthesis pathways, which may help them endure the osmotic stress-induced cellular damage often encountered in marine environments [64]. Since most members of the genus were isolated from marine environments, other pathways associated with marine adaptation were also examined. To handle osmotic stress, bacteria accumulate compatible solutes, which are critical for maintaining cellular integrity and function under hyperosmotic conditions [63,65]. Betaine is a key compatible solute known for its protective role in bacterial osmoregulation. The current analysis revealed that 14 *Microbulbifer* strains lack the complete pathway for betaine biosynthesis (M00555). This absence suggests that these strains might employ alternative strategies in high-salinity marine habitats. Furthermore, bacteria depend on essential antioxidant enzymes, such as superoxide dismutase, catalase, glutathione, and cytochrome oxidase, to neutralize harmful free radicals [66]. All strains retained complete cytochrome c oxidase pathways (M00155 and M00156), which are vital for aerobic respiration under fluctuating oxygen levels, while pathways for cytochrome o oxidase (M00417) and cytochrome bd ubiquinol oxidase (M00153) showed strain-specific distribution, indicating niche-specific adaptations in electron transport (Figure 5). Conversely, the widespread presence of glutathione biosynthesis (M00118) across all 32 strains emphasizes its essential role in combating oxidative stress caused by marine conditions (Figure 5). Additionally, the phosphatidyl ethanolamine (PE) biosynthesis pathway (M00093) was annotated in all *Microbulbifer* strains, as is consistent with the phenotypic test of polar lipids (Table 1). Overall, the analysis of metabolic features and KEGG pathways within the genus *Microbulbifer* reflects their evolved adaptive diversification in response to diverse habitats.

### 3.6. Comparative Analysis of Carbohydrate-Active Enzymes of the Genus Microbulbifer

**Figure 6:**
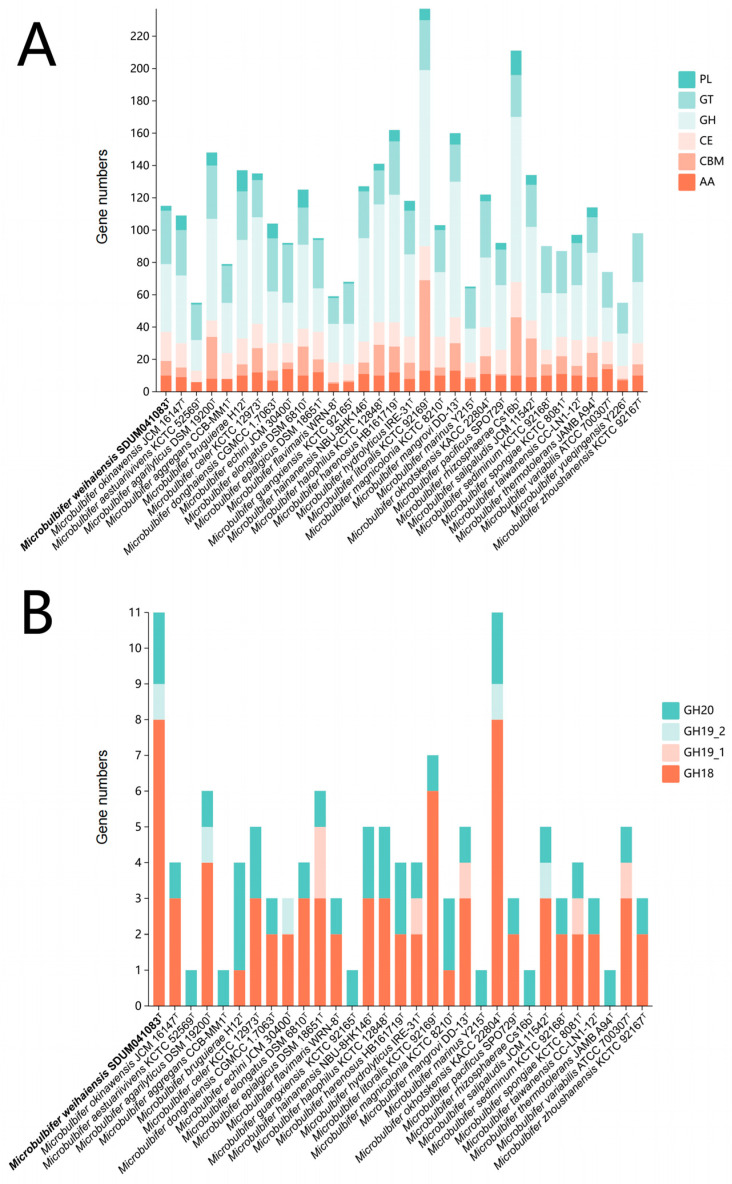
CAZyme family distributions in *Microbulbifer* strains. (**A**) Quantities and distributions of 6 CAZyme families (PL, GT, GH, CE, CBM, AA) across 32 *Microbulbifer* genomes. PL: Polysaccharide Lyases; GT: Glycosyl Transferases; GH: Glycoside Hydrolases; CE: Carbohydrate Esterases; CBM: Carbohydrate-Binding modules; AA: Auxiliary Activities. (**B**) 31 strains with chitin-related CAZymes, showing the presence of 4 chitin-degrading CAZyme families (GH18, GH19_1, GH19_2, GH20). These families are key for chitin degradation, reflecting the genus’s polysaccharide-processing potential.

Building upon prior investigations demonstrating the polysaccharide utilization capabilities of *Microbulbifer* [5,11,12], an assessment of carbohydrate-active enzymes (CAZymes) across 32 strains was conducted (Figure 6A). Different from the research of Long et al. [23], this collection encompassed 31 validly published *Microbulbifer* type strains and the novel strain SDUM041083ᵀ. CAZyme abundance varied considerably (55–237 enzymes), with glycoside hydrolases (GHs) consistently dominating (e.g., >50% in *M. mangrovi*) (Figure 6A), aligning with the genus’s known degradative roles [5,11,12,23]. The proportional composition of these enzyme classes varied across individual strains. This enzymatic profile reflects the *Microbulbifer* genus’s adaptation to multiple ecological niches, such as gut microbiota [55] and aquatic environments [5,7,67], and underscores its evolutionary specialization in key metabolic roles like algal polysaccharide utilization [12] and chitin-degrading [11].

The novel strain SDUM041083^T^ contains 115 annotated CAZymes, mainly including glycoside hydrolases (GHs, 42), glycosyltransferases (GTs, 33), carbohydrate esterases (CEs, 18), auxiliary activities (AAs, 10), and polysaccharide lyases (PLs, 3) (Figure 6B). The identified PLs (PL1_1, PL1_5, PL10_1) suggest pectin lyase activity, while families associated with alginate lyase (PL5/PL7) were absent, aligning with biochemical tests that showed no alginate degradation (Section 3.1). This finding provides genomic insight into the observed enzymatic deficiency. Genomic analysis revealed exceptional chitinolytic potential in SDUM041083ᵀ, featuring 8 GH18, 1 GH19_2, and 2 GH20 genes, representing the highest chitinase gene count among analyzed genomic chitinolytic *Microbulbifer* strains, a trait shared only with *M. okhotskensis* KACC 22804ᵀ [68]. The genomic prediction of robust chitinolytic potential was phenotypically validated through both hydrolysis zone formation (Section 3.1, Figure S2) and the detection of N-acetyl-glucosaminidase activity (GH20 function; Table 1) in biochemical assays. The plate-based assay unequivocally demonstrates high chitinolytic efficacy per unit biomass (H/C ratio), aligning with genomic predictions. Despite its extensive genetic capacity (eight GH18, one GH19_2, and two GH20 genes), the strain exhibits constrained extracellular hydrolytic performance as evidenced by smaller hydrolysis zones compared to *M. okinawensis*. This result may be partially attributed to resource allocation trade-offs favoring chitinase production (or activity) over rapid growth in monotrophic media [69], regulatory constraints requiring chitin oligomers for full induction [70], microbial synergistic degradation [70], substrate specificity of different chitinases [71], and limitations in culture conditions. Consequently, while genomic data confirm the presence of chitin-degrading machinery, its ecological role is likely contingent upon induction conditions specific to its habitat. Future transcriptomic profiling under chitin amendment and enzyme kinetic characterization will elucidate regulatory mechanisms.

Isolated from aquaculture sediment, strain SDUM041083ᵀ possesses genomic features indicating adaptive potential for chitin-rich environments, supported by the plate assay validation of chitin degradation. This suggests a plausible ecological role in marine niches such as aquaculture systems. However, direct ecological niche adaptation requires the isolation of site-specific metagenomic or transcriptomic validation.

### 3.7. Global Biogeographic Distribution of Microbulbifer Based on MAPseq Meta-Analysis

**Figure 7:**
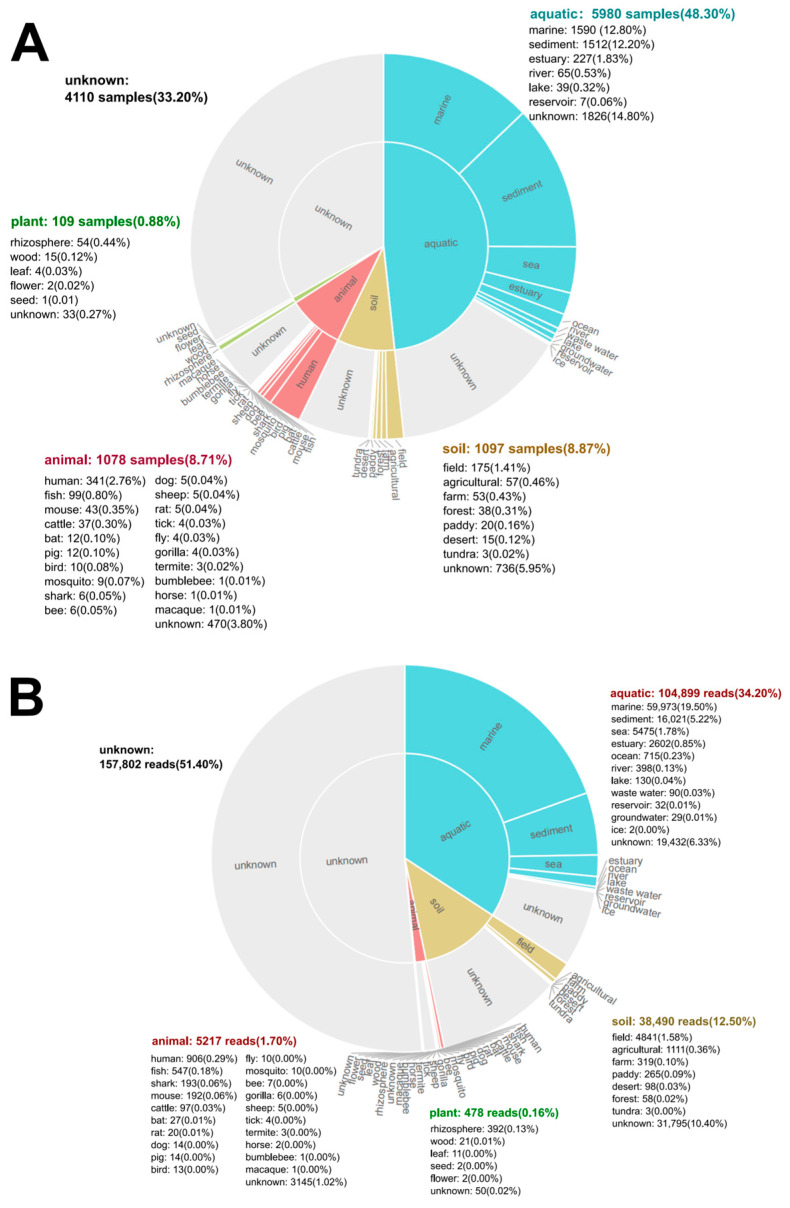
Biogeographic distribution analysis of the genus *Microbulbifer*. (**A**) Frequency of samples with representative OTU sequence, by habitat and sub-habitat. (**B**) Abundance of sequencing reads mapping to the representative OTU sequence, by habitat and sub-habitat.

To comprehensively elucidate the global prevalence and habitat range of the genus *Microbulbifer*, an analysis of this genus’s distribution was performed using the MAP database online. An investigation was conducted on 12,374 samples spanning 2326 projects to pinpoint the characteristic sequence. *Microbulbifer* sequences were detected in 5980 aquatic (48.30%), 1097 soil (8.87%), 1078 animal (8.71%), and 109 plant samples (0.88%) (Figure 7A). Within aquatic environments, marine habitats (12.80%) and sediments (12.20%) showed the highest prevalence. Furthermore, a comparative analysis of database sequence reads against standard OTUs revealed that *Microbulbifer* is predominantly enriched in marine environments (19.50% of reads), with sediment environments representing the secondary niche (5.22%) (Figure 7B). This genus-level distribution aligns with the marine origins of most cultured *Microbulbifer* strains [23].

Critically, as emphasized above, the biogeographic distribution inferred from MAPseq (based on 16S rRNA gene profiling) reflects genus-level patterns and cannot attribute strain-specific functional traits, such as the chitinolytic proficiency of SDUM041083^T^. *Microbulbifer*’s broad environmental prevalence likely stems from metabolic conservation (e.g., osmotic stress amino acid synthesis; Section 3.5), CAZyme-mediated niche plasticity enabling polysaccharide versatility (Figure 6), and lineage-specific adaptations (e.g., betaine-independent osmoregulation) recurrent in coastal *Microbulbifer* strains, including SDUM041083^T^ and 13 conspecific isolates (Section 3.5). While these traits facilitate habitat colonization, strain-level ecological roles require functional validation beyond 16S data.

### 3.8. Description of Microbulbifer weihaiensis sp. nov.

The cells are Gram-negative, motile, straight-rod shaped (4.780 ± 0.2 μm × 0.326 ± 0.1 μm), aerobic, oxidase-negative, and peroxidase-positive, and they form yellow circular colonies. Cells can grow on MA medium, and Na^+^ must be present for growth. Cell growth on MA can occur at temperatures ranging from 25 to 40 °C, with 35 °C being the optimal temperature for growth. Additionally, 2% (*w*/*v*) NaCl was the optimal condition for growth, and cells can grow on a medium containing 1–10% (*w*/*v*) NaCl, but growth was very slow at 7–10% salinity. The pH at which the cell could grow ranged from 5.5 to 9.5, with 7.0 being the optimal pH. The cells were able to hydrolyze Tween 20, 40, 60, and 80 and showed caseinase activity, but they did not respond to alginate, cellulose, agar, or DNA. Nitrate metabolism was positive. In the API ZYM system, alkaline phosphatase, esterase (C4), lipoid esterase (C8), lipase (C14), leucine arylamines, valine arylamines, cystine arylaminase, pancreatic coagulase, acid phosphatase, naphthol-AS-BI-phosphohydrolase, alpha-glucosidase, and N-acetyl-glucosaminidase were positive (intensities 3–5), while the rest were negative. In the API 20E system, the pyruvate, gelatinase, glucose, and arabinose test results were positive, but the remainder produced negative results. Q-8 was the only respiratory quinone. The main fatty acids (>10.0%) were iso-C_15:0_ (24.33%), iso-C_11:0_ 3-OH (10.78%), and iso-C_11:0_ (10.61%). The major polar lipids were phosphatidylglycerol (PG), phosphatidylethanolamine (PE), and aminolipid (AL).

The type strain was SDUM041083^T^ (= MCCC 1H01537^T^= KCTC 8896^T^), which was isolated from the shoal sediment of Xiaoshidao, Weihai, China. The DNA G+C content of this type strain was 57.5 mol%. The GenBank accession number for the 16S rRNA gene sequence of *Microbulbifer weihaiensis* SDUM041083^T^ is PV225712. This Whole Genome Shotgun project has been deposited at DDBJ/ENA/GenBank under the accession JBLWFL000000000.

## Figures and Tables

**Table 1:** Differential characteristics of SDUM041083^T^ and closely related species. Strains: 1, SDUM041083^T^; 2, *M. okinawensis* JCM 16147^T^; 3, *M. echini* JCM 30400^T^; 4, *M. yueqingensis* JCM 17212^T^. +, Positive; −, negative; (+), weakly positive reaction. All strains are Gram-stain-negative, aerobic, and contain Q-8 as the major respiratory quinone. All of the data were from this study unless otherwise specified. Polar lipids, unidentified and unknown are not mentioned. Data from: ^a^ ([55]), ^b^ ([54]).

Characteristic	1	2	3 ^a^	4 ^b^
sampling environment	Intertidal sediments	mud samples from mangrove forests	the gastrointestinal tract of a purple sea urchin	sediment
Motility/flagella	+/−	−/−	−/−	−/−
Temperature range (optimum) (°C)	20–40 (35)	10–45 (37)	10–36 (30)	15–45 (30–37)
NaCl range (optimum) (g/L)	5–110 (35)	5–150 (30)	10–80 (20)	0–100 (20–30)
pH range (optimum)	5.5–9.5 (7.0)	5.5–9.5 (7.0–7.5)	6.2–9.0 (7.0)	5.0–10.0 (7.0–8.0)
DNA G+C content (mol%)	57.5	57.8	56.1	56.7
Polar lipids	PG, PE, AL	PG, PE	PE, PS	PE, PG
Oxidase	−	+	+	+
Hydrolysis of:				
Tween-20	+	+	−	−
Tween-40	+	+	+	−
Casein	+	+	+	+
Starch	−	+	+	−
Gelatin	+	+	+	−
API ZYM tests:				
Cystine arylamines	+	+	−	−
chymotrypsin	+	−	−	−
N-acetyl-glucosaminidase	+	+	−	−
Trypsin	−	−	−	+
lipase (C14)	(+)	+	+	−

## Data Availability

The GenBank accession number of *Microbulbifer weihaiensis* SDUM041083^T^ for the 16S rRNA gene sequence is PV225712; for the whole-genome assembly, it is JBLWFL000000000. The BioProject accession number of *Microbulbifer weihaiensis* SDUM041083^T^ is PRJNA1226674.

## Supplementary Materials

The following supporting information can be downloaded at https://www.mdpi.com/article/10.3390/microorganisms13092005/s1, Figure S1: Scanning electron micrograph of cells of strain SDUM041083^T^. Bar, 2 μm; Figure S2: The chitin-degrading hydrolysis zone of strain SDUM041083^T^. The left half of the plate is SDUM041083^T^, and the right half is the reference strain *M. okinawensis* JCM 16147^T^; Figure S3: Two-dimensional TLC plate image of the total polar lipids of strain SDUM041083^T^ (a) and *M. okinawensis* JCM 16147^T^.PE, phosphatidylethanolamine; AL, unidentified aminolipid; PG, phosphatidylglycerol; Figure S4: The distribution of core genes, accessory genes, and unique genes to different metabolic pathways in the genus *Microbulbifer*; Table S1: Cellular fatty acids composition (%) of the strain SDUM041083^T^ and relative strains: 1, SDUM041083^T^; 2, *M. okinawensis* JCM 16147^T^; 3, *M. taiwanensis* CCM 7856^T^. Only those fatty acids accounting for 1% or more in one of the strains are given. tr, trace amount (<1.0%); −, not detected.
