# Supplementary material for: Analysis of Genomic and Characterization Features of Microbulbifer weihaiensis sp. nov., Isolated from Coastal Sediment
Source: Microorganisms. 2025 Aug 28;13(9):2005. doi: 10.3390/microorganisms13092005 (PMC12472934; doi:10.3390/microorganisms13092005)
Supplement: Supplementary file 1 [file microorganisms-13-02005-s001.zip › microorganisms-3804563-supplementary.pdf]

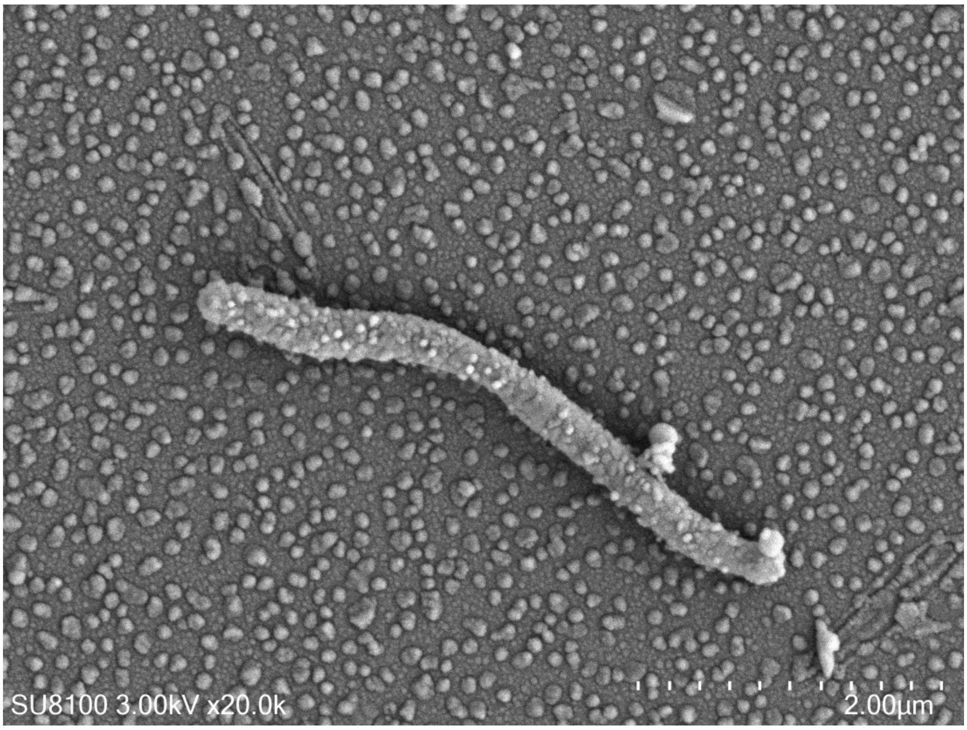

Figure S1. Scanning electron micrograph of cells of strain SDUM041083^T. Bar, 2 μm.

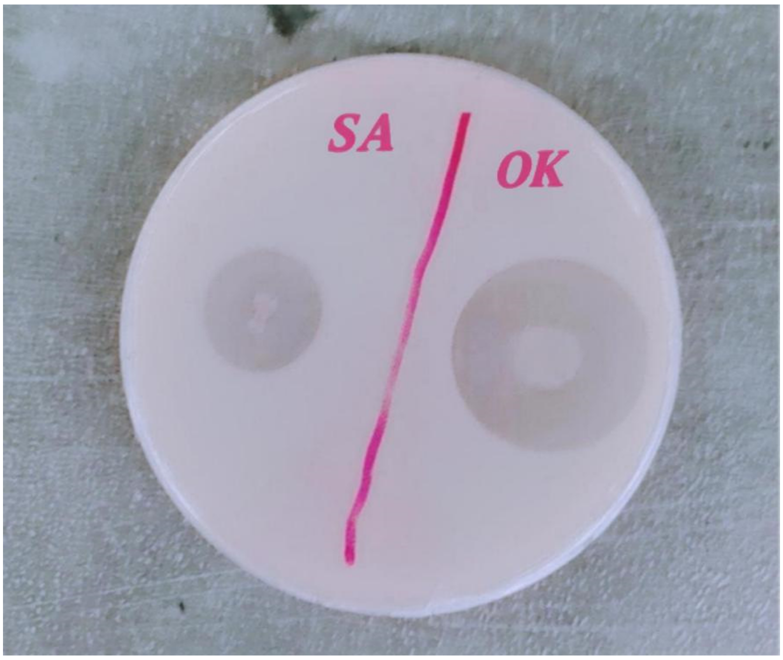

Figure S2. Chitin-degrading hydrolysis zones of strain SDUM041083^T and the positive control. The left half of the colloidal chitin plate (SA) is SDUM041083^T, and the right half (OK) is the positive control strain *M. okinawensis* JCM 16147^T. Plates were incubated at 35°C for 7 days. Quantitative analysis shows the ratio of hydrolysis zone diameter to colony diameter: 5.25 ± 0.10 for SDUM041083^T and 2.86 ± 0.10 for *M. okinawensis* JCM 16147^T.

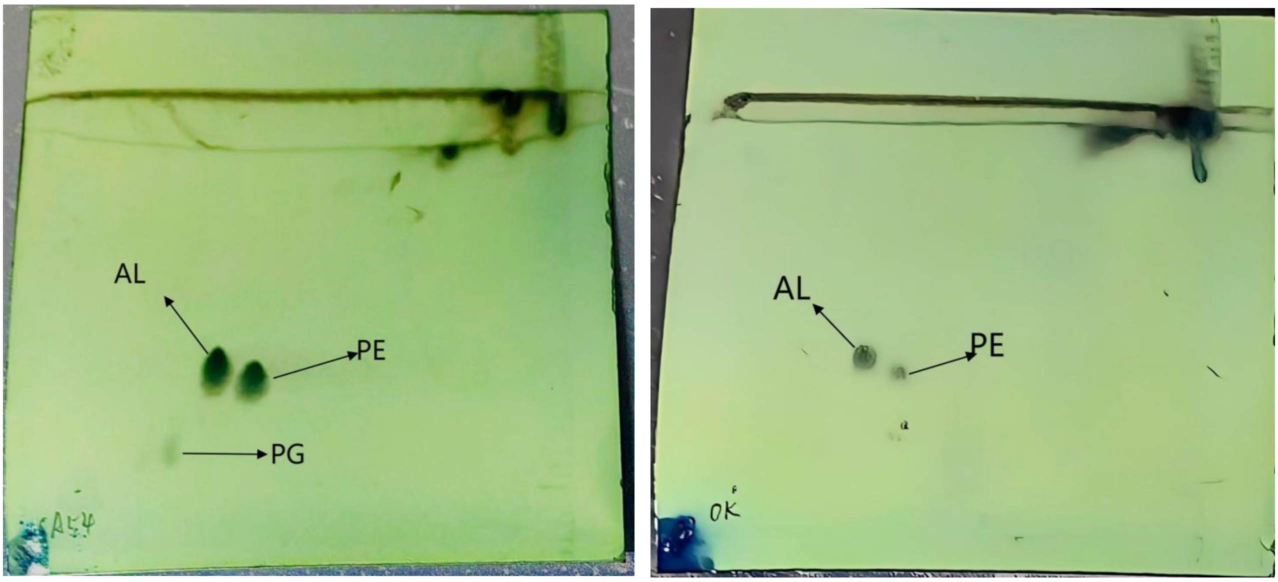

(a) (b)

Figure S3. Two-dimensional TLC plate image of the total polar lipids of strain SDUM041083^T (a) and *M. okinawensis* JCM 16147^T (b). PE, phosphatidylethanolamine; AL, unidentified aminolipid; PG, phosphatidylglycerol.

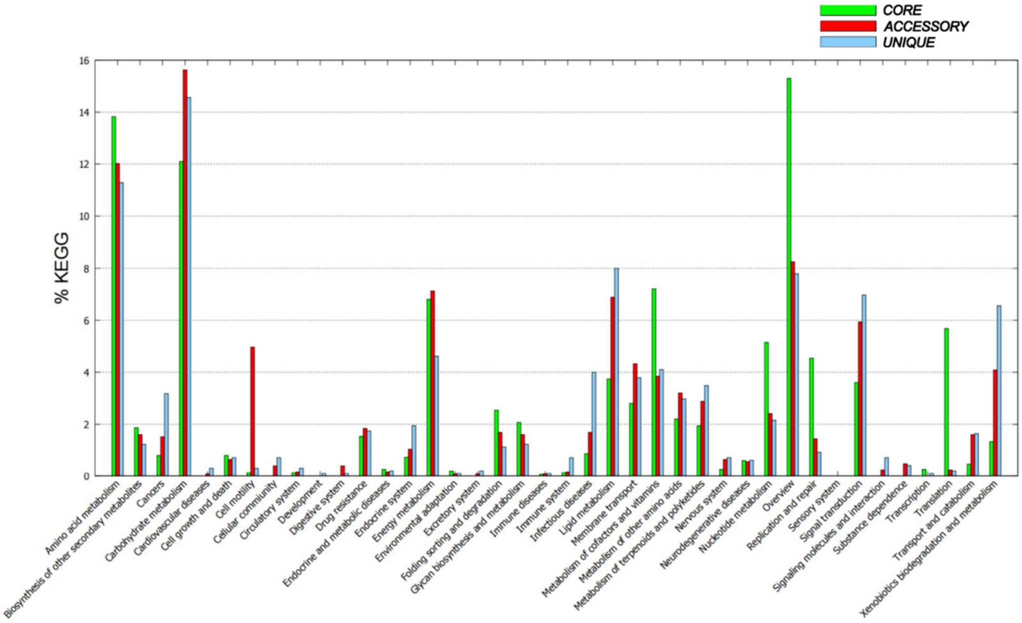

Figure S4. The distribution of core genes, accessory genes and unique genes to different metabolic pathways in the genus *Microbulbifer*.

Table S1. Cellular fatty acid composition (%) of the strain SDUM041083^T and relative strains: 1, SDUM041083^T; 2, *M. okinawensis* JCM 16147^T; 3, *M. taiwanensis* CCM 7856^T. Only those fatty acids accounting for 1 % or more in one of the strains are given. tr, trace amount (<1.0%); −, not detected.

Fatty acid	SDUM041083^T	*M. okinawensis*	*M.taiwanensis*
Straight-chain fatty acids			
$C_{10:0}$	tr	tr	tr
$C_{14:0}$	tr	tr	-
$C_{16:0}$	3.27	4.41	2.3
$C_{17:0}$	2.29	2.44	1.1
Branched fatty acids			
iso-$C_{11:0}$	10.61	8.61	5.3
iso-$C_{13:0}$	-	-	-
iso-$C_{15:0}$	24.33	28.71	22.6
iso-$C_{15:1}$ F	1.09	1.88	3.1
iso-$C_{17:0}$	8.13	7.99	5
Hydroxy fatty acids			
$C_{10:0}$ 3-OH	tr	tr	1.0
iso-$C_{11:0}$ 3-OH	10.78	8.29	10.4
$C_{11:0}$ 3-OH	tr	tr	-
iso-$C_{15:0}$ 3-OH	tr	tr	-
Unsaturated fatty acids			
$C_{15:1}$ ω8c	tr	tr	-
$C_{15:1}$ ω6c	tr	tr	1.4
$C_{15:1}$ ω5c	2.48	2.32	-
$C_{17:1}$ ω7c	–	–	-
$C_{17:1}$ ω6c	–	–	-
$C_{17:1}$ ω8c	tr	1.60	2.5
cyclo-$C_{17:0}$	4.48	2.01	-
